# Spatial Transmission Characteristics of the Bluetongue Virus Serotype 3 Epidemic in The Netherlands, 2023

**DOI:** 10.3390/v16040625

**Published:** 2024-04-17

**Authors:** Gert-Jan Boender, Thomas J. Hagenaars, Melle Holwerda, Marcel A. H. Spierenburg, Piet A. van Rijn, Arco N. van der Spek, Armin R. W. Elbers

**Affiliations:** 1 Department of Epidemiology, Bioinformatics, Animal Studies and Vaccine Development, Wageningen Bioveterinary Research, P.O. Box 65, 8200 AB Lelystad, The Netherlands; gertjan.boender@wur.nl (G.-J.B.); thomas.hagenaars@wur.nl (T.J.H.); 2 Department of Virology, Wageningen Bioveterinary Research, P.O. Box 65, 8200 AB Lelystad, The Netherlands; melle.holwerda@wur.nl (M.H.); piet.vanrijn@wur.nl (P.A.v.R.); 3 Incident- and Crisis Centre (NVIC), Netherlands Food and Consumer Product Safety Authority (NVWA), P.O. Box 43006, 3540 AA Utrecht, The Netherlands; m.a.h.spierenburg@nvwa.nl (M.A.H.S.); a.n.vanderspek@nvwa.nl (A.N.v.d.S.); 4 Department of Biochemistry, Centre for Human Metabolomics, North-West University, Private Bag X 6001, Potchefstroom 2520, South Africa

**Keywords:** bluetongue, BTV-3, spatial kernel, spatial spread, transmission

## Abstract

A devastating bluetongue (BT) epidemic caused by bluetongue virus serotype 3 (BTV-3) has spread throughout most of the Netherlands within two months since the first infection was officially confirmed in the beginning of September 2023. The epidemic comes with unusually strong suffering of infected cattle through severe lameness, often resulting in mortality or euthanisation for welfare reasons. In total, tens of thousands of sheep have died or had to be euthanised. By October 2023, more than 2200 locations with ruminant livestock were officially identified to be infected with BTV-3, and additionally, ruminants from 1300 locations were showing BTV-associated clinical symptoms (but not laboratory-confirmed BT). Here, we report on the spatial spread and dynamics of this BT epidemic. More specifically, we characterized the distance-dependent intensity of the between-holding transmission by estimating the spatial transmission kernel and by comparing it to transmission kernels estimated earlier for BTV-8 transmission in Northwestern Europe in 2006 and 2007. The 2023 BTV-3 kernel parameters are in line with those of the transmission kernel estimated previously for the between-holding spread of BTV-8 in Europe in 2007. The 2023 BTV-3 transmission kernel has a long-distance spatial range (across tens of kilometres), evidencing that in addition to short-distance dispersal of infected midges, other transmission routes such as livestock transports probably played an important role.

## 1. Introduction

Bluetongue (BT) is a non-contagious, arthropod-borne viral disease of domestic and wild ruminants, and the transmission between hosts depends on competent *Culicoides* midge species [1]. Historically, BT was not endemic in Europe, but infrequent incursions occurred into this area. Since 1998, BT has been established in Southern Europe and the Mediterranean Basin and is caused by several serotypes of bluetongue virus (BTV) [2]. These BTV serotypes have likely been introduced by legal (and possibly illegal) live animal trade and/or the wind-driven dissemination of infected *Culicoides* midges through three main corridors: via Turkey, Greece, and the Balkan; via North Africa to Spain through the Strait of Gibraltar; and to Italy via Sicily or Sardinia [3].

The first ever recorded introduction of BTV in the Netherlands occurred in 2006. It was a serotype-8 (BTV-8) strain, and its introduction was followed by a large epidemic extending across Belgium, the Netherlands, and most of France and Germany in 2007 [4]. Temporally, this epidemic took place across the vector-active season in 2006 followed by further epidemic spread during the vector-active season of 2007 [5]. In addition, in late 2008, a vaccine-related BTV-6 was detected in animals from 18 livestock herds by PCR in the Netherlands. This BTV-6-strain was not efficiently transmitted by the endemic species of *Culicoides* midges in Northwestern Europe and disappeared without the need of any control measures [6].

The epidemic in the Netherlands in 2023 was associated with the BTV serotype 3 (BTV-3). Clinical signs observed in sheep, and typical for BT, led to the confirmation of BTV-3 infection in the beginning of September 2023 [7] and marked the start of a fast-expanding epidemic within the Netherlands, which subsequently spread to Belgium, Germany, and Great Britain [8]. Historically, the basis of BTV serotype 3 (BTV-3) strains can be divided into at least two main clusters [9]: (i) a cluster of strains originating from Africa, the Mediterranean Basin, and North America (western topotypes) and (ii) a cluster of strains originating from Japan, India, and Australia (eastern topotypes). Recently, BTV-3 outbreaks were reported in Italy in 2017–2022 [10,11,12]. BTV-3 has also been reported in Tunesia and Israel [9,13]. The genomic sequence of the viral protein 2 (VP2) of BTV-3/NET2023 has the highest homology with strains from Europe, indicative of a potential western topotype [7]. Further, full genome sequencing has shown that BTV-3/NET2023 which is causing the current BT-3 outbreak in the Netherlands is significantly different from known and published BTV-3 strains [7].

Livestock diseases transmitted by biting insects can be spread to other farm locations via the movement of infected livestock and the dispersal of infected vectors. We can restrict the movement of livestock hosts, and with that potentially reduce the pace at which the epidemic spreads, but we cannot limit the diffusion of infected midges. A review by Elbers et al. [14] concluded that individual *Culicoides* midges, within just a few days, are able to traverse distances of up to 5 km. This flight range shows *Culicoides* to be capable flyers, and in line with the observation that the lure of livestock causes them to disperse from farm-to-farm and pasture-to-pasture due to the associated presence of livestock within any farming environment [15,16]. It is not clear to what extent the local movements depend on wind assistance. Wind assistance can be involved in less frequent long-distance (coincidental) movement of midges, which supports migration [13]. This long-distance movement is most efficiently achieved by rapid winds leading to their spread over tens to hundreds of kilometres, especially over the sea, and that may result in incursions in distant areas [14].

The between-farm transmission dynamics of the 2006–2007 BTV-8 epidemic in Northwestern Europe has been analysed by several authors, most of whom applied a between-farm transmission kernel approach [5,17,18]. This approach models the between-farm transmission without distinguishing the contribution of different transmission routes such as over-the-fence, through midge dispersal, and through animal movement. Instead, it only models the total transmission hazard, and achieves this by means of a distance-dependent transmission kernel. Szmaragd et al. [17] and De Koeijer et al. [18] analysed the BTV-8 data for 2006 and Boender et al. [5] investigated the data for 2007. These analyses showed some consistency between the estimated BTV transmission kernels in the different years and areas. In particular, in the study by Boender et al. [5], it was found that the transmission kernel for 2007, as estimated across all areas of France, Germany, and the Netherlands that were not yet affected in 2006, was similar to the transmission kernel based on the 2006 outbreak data for Germany. The between-farm transmission hazard declined very slowly with distance between an infectious and a susceptible farm: only at approximately 18 to 23 km distance, this hazard had declined to half of its value at ‘zero distance’ in the analyses. Similarly, long transmission ranges were found in the analyses of Szmaragd et al. [17] despite having technical differences with those obtained from the kernel estimation approach used by de Koeijer et al. [18]. Here, we present a transmission kernel analysis for the between-farm spread of BTV-3 in the Netherlands in the period between 31 August (the date of collection of the first confirmed BTV-3-positive sample) and 17 October 2023. Further, we compared these results with the transmission kernels estimated earlier for BTV-8 transmission in Northwestern Europe in 2006 and 2007.

## 2. Materials and Methods

*Denominator data*: This dataset was obtained from Netherlands Enterprise Agency (Rijksdienst voor Ondernemend Nederland, RVO) and contains information on 31,130 registered farm locations with cattle, sheep, and/or goats in the Netherlands, including farm location identification number (farm location ID), spatial coordinates, number of animals, and animal type.

*Dataset of BTV cases based on PCR results:* This dataset was constructed based on confirmed PCR test results of the clinical suspicions of BTV infection submitted to Wageningen Bioveterinary Research (WBVR) between 31 August 2023 and 17 October 2023. This dataset contains 1601 sample shipments, each one containing one or more samples from the same farm location. For 187 shipments, no farm location ID was provided. The remaining 1414 shipments with farm location IDs together contained 1375 unique farm locations. In detail, a total of 1339 farm locations submitted one shipment, 34 locations submitted two shipments, 1 location submitted three shipments, and 1 location submitted four shipments. As a result, the dataset contains 1375 farm locations that tested PCR positive for BTV-3. We named these locations ‘positive case locations’.

*Dataset of BTV cases based on clinical diagnosis:* In addition, cases were reported based on clinical diagnosis. This dataset contains 702 reports. Of these reports, 19 did not include a farm location ID. As none of these 19 reports could be linked through the address to a farm location ID in the denominator data, all 19 were removed. Taking also into account that there were 11 farm locations with two reports, this led to 672 ‘clinical case locations’.

*Dataset for analysis*: A dataset for analysis was constructed by linking the data on the case locations to the denominator dataset as follows. A total of 947 positive case locations were linked through the identification number. The remaining 428 (=1375–947) positive case locations had an identification number that was not present in the denominator dataset. For these 428 locations, spatial coordinates could be assigned to 426 locations based on the address, while for the remaining 2 locations, this was not possible.

Of the 672 clinical case locations, 9 were already present in the dataset of positive case locations. From the remaining 663 clinical case locations, 219 were not present in the denominator dataset, and spatial coordinates were assigned based on the address.

**Figure 1:**
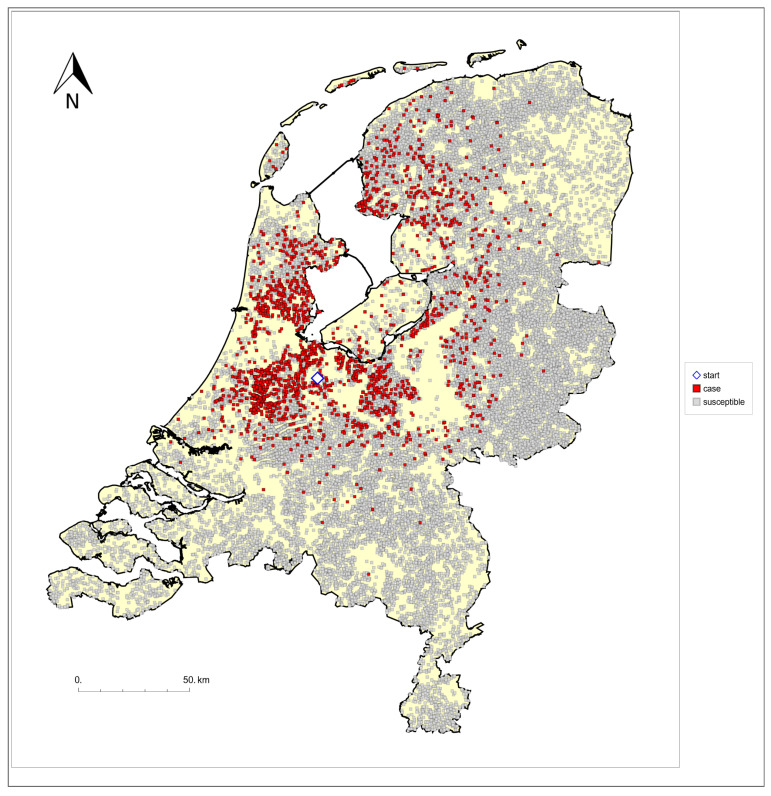
Map of the location of all BTV-3 outbreak farms (red dots) included in the analysis; the farm detected first is indicated by a diamond. The grey dots are locations with ruminants not confirmed as affected farm by mid-October.

Overall, this led to a crude dataset of 2055 case locations (positive and clinical), and a total denominator population of 31,775 locations. The vast majority of these were locations with cattle, sheep, and/or goats. In addition, arising from the case data, there were nine locations with other species susceptible to BTV (such as Alpaca and Llama). As different animal types on the same location may be registered with different farm location IDs, in a further step, multiple occurrences of one and the same farm location were combined into one, which involved, among others, six case locations being counted twice. This led to a final dataset for analysis that contained 30,993 farm locations, including 2049 case locations (=2055–6 case locations). For all case locations, the dataset contained a date of clinical suspicion. We refer to these cases below as outbreaks or outbreak farms. The locations of these outbreak farms are shown in Figure 1. As can be seen in Figure 1, the main pattern of spread until mid-October did not extend to or beyond the Dutch borders. As a result, the use of only Dutch denominator data, i.e., ignoring potentially exposed farms across the borders, is not expected to introduce an important bias in our quantifications of the distance-dependent transmission. In Figure 1, the first detected outbreak is indicated with a diamond. It can be seen that, from the initial outbreaks in the centre of the Netherlands, the spatial epidemic spread occurred mostly in the eastern, western, and northern directions, and to a lesser extent, in the southern direction.

### Modelling

Between-farm transmission is represented by a transmission kernel $h(r_{ij})$, which is only a function of the Euclidean (i.e., straight-line) distance $r_{ij}$ between the farms. This transmission kernel describes the hazard $\lambda_{i}$ with which a susceptible farm becomes infected on day t as follows:

$$\lambda_{i}\left(t\right)=\sum_{j}h\left(r_{ij}\right),$$

with j running over all infectious farms at day t. The transmission kernel h(r) is usually assumed to be a non-increasing function of r, governed by a limited number of parameters. In line with the earlier studies [5,18], we use the ‘Cauchy’ form of the transmission kernel:

(1) $$\lambda \left(r\right)=\frac{\lambda_{0}}{1+{\left(\frac{r}{r_{0}}\right)}^{\alpha }}$$

in which r is the straight-line distance between an infectious and a susceptible farm. The parameter $\lambda_{0}$ represents the amplitude of the transmission kernel and is equal to the value of the transmission hazard for a very small distance (‘distance zero’) between the infectious and the susceptible farm. As a simplifying assumption, $\lambda_{0}$ is taken to be time-independent, i.e., independent of calendar time (and also independent of how much of the infectious period of the infectious farm has elapsed). As a consequence, when estimated from observations, the value for $\lambda_{0}$ represents a time-averaged kernel amplitude over the period of the observations (from 31 August to 17 October 2023). The parameters $r_{0}$ and α together determine the shape of the transmission kernel, i.e., they are termed as the shape parameters. The parameter $r_{0}$ is a characteristic distance, also referred to as ‘kernel offset’ [19]. It is the distance where the transmission hazard has become half as large as at distance zero. The parameter α is a scaling exponent that determines how fast the transmission hazard declines for longer distances, and its influence on the kernel shape dominates over the influence of $r_{0}$ for distances a few times larger than $r_{0}$ and beyond. To estimate the (parameters of the) transmission kernel (1), we followed the same procedure as followed by De Koeijer et al. [18]. This analysis requires an estimated day of infection and an infectious period for each outbreak farm. In accordance with the approach followed earlier [5,17], the estimated day of infection was set at two weeks before the day of suspicion, while the starting day of the infectious period was identified with the day of suspicion as listed in the dataset. These assumptions are presented and motivated in [5,18] and are broadly in line with experimental results for individual animals [20]. As no interventions such as culling or vaccination were applied, all outbreak farms were assumed to remain infectious until the end of the period studied (mid-October 2023). Based on the days of infection and infectious periods, a list of possible transmission events and of ‘escape events’ was composed. An escape event is defined as a susceptible farm escaping from infection for one day. The list of possible transmission events contains for each outbreak farm the list of distances to all the locations that were infectious on the day of infection of the outbreak farm, and therefore could be the infector of this outbreak farm. The list of escape events contains for each (outbreak farm, escape day) combination, the list of distances to all the locations that were infectious. Here, as ‘escape day’, all days are included on which the farm still escaped from infection. It also contains for each (escape farm, escape day) combination, the list of distances to all the locations that were infectious. Here, an ‘escape farm’ is a farm that escaped from infection until 17 October 2023, and as ‘escape day’, all days between 31 August and 17 October are included.

Using these lists and Equation (1), the model parameter likelihood can be computed. In a discrete-time approximation with timestep ∆t=1d (one day), this likelihood is given by the following expression:

$$L=\prod_{i}P_{\mathrm{esc},i}\left(t_{\inf,i}\right)P_{\inf,i}\left(t_{\inf,i}\right)\prod_{j}P_{\mathrm{esc},j}\left(t_{\mathrm{end}}\right)$$

where i runs over all farms that became infected during the epidemic, and j runs over all farms that escaped from (detected) infection throughout the epidemic. The day when the infectiousness of the first infected farm was assumed to start (i.e., 31 August) was defined as day 1 in this analysis. Day $t_{\mathrm{end}}$ denotes the last day of the period of study (i.e., $t_{\mathrm{end}}=$ 48, corresponding to 17 October), and $P_{\mathrm{esc},i}(t)$ denotes the probability of escaping infection until day t:

$$P_{\mathrm{esc},i}(t)\equiv \exp\left(-\sum_{\tau =1}^{t-1}\lambda_{i}\left(\tau \right)∆t\right),$$

and $P_{\inf,i}(t)$ denotes the probability that farm i acquires infection during the t-th day:

(2) $$P_{\inf,i}\left(t\right)\equiv 1-\exp\left({-\lambda }_{i}\left(t\right)∆t\right)$$

**Figure 2:**
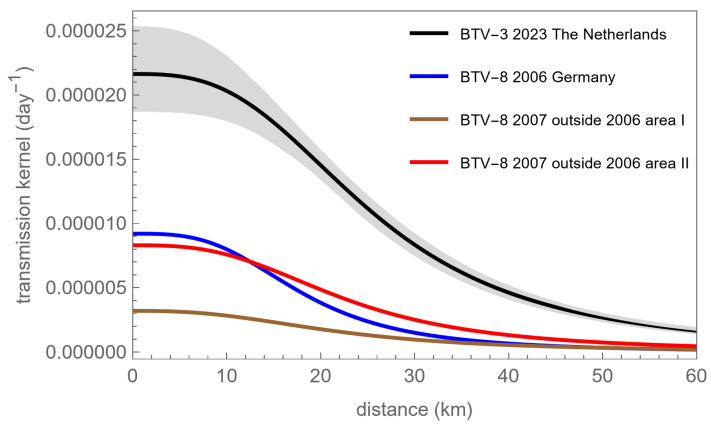
Spatial transmission kernel estimated for BTV epidemic datasets. The kernels are estimated for the BTV-3 2023 dataset (black line and grey confidence areas), the BTV-8 2007 dataset in which the BTV-8 2006 epidemic area is removed by taking out a circle of 200 km radius (brown line, data from De Koeijer et al. [18]), the BTV-8 2007 dataset in which the BTV-8 2006 epidemic area is removed by taking out circles of 80 km radius around each case of the 2006 epidemic (red line, data from Boender et al. [5]), and the 2006 German dataset (blue line, data from De Koeijer et al., [18]).

The parameters of the transmission kernel enter the likelihood L through $\lambda_{i}$ and are estimated by maximizing L (maximum likelihood estimation). The corresponding univariate 95% confidence bounds are obtained using the likelihood-ratio test. For these analyses and for the visualisations in Figure 1 and Figure 2, we used purpose-written software coded in Mathematica [21].

## 3. Results

In Table 1, we list the point estimates of the kernel parameters and their confidence bounds. As a reference, we also include estimates obtained by De Koeijer et al. [18] and by Boender et al. [5] for three different periods and/or areas in the BTV-8 epidemic of 2006–2007. In Figure 2, the kernels corresponding to the estimates in Table 1 are plotted.

As seen in Table 1 and Figure 2, the kernel for BTV-3 in NL in 2023 is found to have a shape similar to the Europe-2007 kernel variants and the German-2006 kernel variants, i.e., for both shape parameters $r_{0}$ and α, the values found are very similar across all four epidemic datasets. All four curves are only slowly decreasing at longer distances, implying that apart from a considerable amount of local spread (to be expected due to midge dispersal), there is also considerable transmission across longer distances. The values listed in Table 1 for the kernel shape parameters $r_{0}$ and α are very similar between the years 2006, 2007, and 2023.

In contrast, the amplitude parameter $\lambda_{0}$ is observed to differ substantially between the different analysed epidemics. Most importantly, it is higher in the Netherlands-2023 analysis than in the Germany-2006 and Europe-2007 analyses, and this is statistically significant as can be observed from the confidence bounds given in Table 1. This difference amounts to a factor of between 2.4 (ratio of Netherlands-2023 to Germany-2006 point estimates of the amplitude parameter $\lambda_{0}$) and 6.9 (ratio of Netherlands-2023 to Europe-2007—version ‘without 2006 infected area’—point estimates of the amplitude parameter $\lambda_{0}$). In addition, in Figure 2, it is observed that the kernels, Germany-2006 and Europe-2007, are well below the grey confidence area of the Netherlands-2023 kernel, showing that the difference between the transmission hazards is significant at all distances.

Our interpretation of the similarity between the kernel shape parameter estimates and of the difference between the amplitude parameter estimates of different epidemics is described in Section 4 (the Discussion Section).

The values given are the maximum likelihood estimates, with the univariate confidence bounds between brackets obtained from the likelihood-ratio test. As explained in more detail in the main text, *λ*_0_ is the transmission hazard for a very small distance (‘distance zero’) between an infectious and a susceptible farm, *r*_0_ is the distance where the transmission hazard has become half as large as at distance zero, and α determines how fast the transmission hazard declines for long distances. Parameter values estimated from the 2007 dataset in which the 2006 epidemic area is removed by taking out an area with a radius of 200 km, and from the 2007 dataset in which the 2006 epidemic area is removed by taking out areas with a radius of 80 km around each case of the 2006 epidemic.

## 4. Discussion

Seventeen years after the 2006 BTV-8 epidemic struck Northwestern Europe, we encountered another emergence of a bluetongue virus in the Netherlands, and this time it was BTV-3.

With respect to environmental temperature, similar conditions were observed in the Fall of 2006 and 2023 and were highly favourable for BTV transmission: average temperatures of 17.9 and 17.5 °C in September; 13.6 and 13.2 °C in October; and 9.2 and 7.8 °C in November, respectively (De Bilt weather station; Source: Royal Netherlands Meteorological Institute, www.knmi.nl). These average monthly temperatures were unusually high: approximately 2 to 3 °C higher than the normal average monthly temperature over the preceding 30 years.

Wind direction data from the Royal Netherlands Meteorologic Institute indicate that, in the study period, for less than 10% of the observation days, wind was blowing in the southern direction, while on the basis of a homogeneous distribution, this would be expected to be around 25%. However, in line with the previous analyses [4,15], we did not attempt to include any dependence on wind direction in our analysis. As a result, we did not investigate to which extent wind direction could explain the fact that the epidemic moved spatially in the eastern, western, and northern directions, but hardly in the southern direction (Figure 1).

Perhaps, the most important result of our analysis is the similarity between the kernel shape parameter estimates of different epidemics. This finding suggests that the distances over which the virus was transmitted in 2023 followed a distribution very similar to 2006 and 2007, providing no evidence for a change in the relative importance of transmission mechanisms provided by the short-distance dispersal of infected midges versus other mechanisms operating at longer distances.

For the interpretation of the difference in the amplitude parameter $\lambda_{0}$ between the different analysed epidemics, it is important to consider that (1) the estimates obtained for $\lambda_{0}$ represent an average across the analysed period; (2) there is a proportionality (to a good approximation) between the amplitude parameter $\lambda_{0}$ and the average (across the analysed period) of the effective between-holding reproduction number R; and (3) in the study by Boender et al. [5], a marked correlation was found between the time-dependent R value and a 14-day average temperature. This correlation is likely to arise due to higher temperatures promoting both the abundance of midges as well as virus replication. Due to this temperature dependence, in combination with the seasonality of temperature, different $\lambda_{0}$ estimates are expected because there are substantial differences in the calendar dates of the analysed periods: The Germany-2006 and Europe-2007 periods of analysis last well beyond the mid-October end of the Netherlands-2023 period. Mainly, as a result of this, the average temperature across the period considered in the Netherlands-2023 analysis is several degrees Celsius higher than for the Germany-2006 and Europe-2007 analyses. In more detail, for the 2023-Netherlands analysis period the average temperature was 16.7 °C when measured by the centrally located weather station at De Bilt. For the Germany-2006 analysis period, it was 14.3 °C (measured by Kassel weather station in Germany), and for the period of the Europe-2007 analyses, we found an average temperature of 14.5 °C when averaging across the weather stations located in De Bilt, Kassel, and Aulnois-sous-Laon (France). Based on the correlation shown by Boender et al. [5], a value between 2.4 and 6.9 for the ratio between the amplitude parameter value for Netherlands-2023 and the value in the earlier analyses is consistent with these differences in average temperature. This means that the difference in amplitude parameter values does not necessarily provide evidence for biological differences between the involved viruses. For example, it does not provide evidence that the BTV-3 strain would have a different interaction with the vector compared to that of the BTV-8 strain of 2006 and 2007, e.g., by having a higher vector competence.

We note that a consequence of a higher vector competence would be a higher proportion of midges that become infected. For example, the prevalence of Schmallenberg virus (SBV) during the epidemic in the Netherlands in 2011 was 0.56% in *Culicoides obsoletus* and *C. scoticus* and 0.14% in *C. chiopterus*, and this was about 10 times and 5 times higher, respectively, than reported earlier for BTV [22]. A higher proportion of infected midges was observed during the SBV epidemic compared to the BTV epidemics and was hypothesized to be an explanation for the faster spread of SBV compared to the spread of BTV-8 in 2006–2008 in the Netherlands [22].

As was noted before [5], the kernel based on the BTV-8 outbreak pattern for the Netherlands in 2006 was found to be quite different from Germany-2006 and the results of the Europe-2007 analysis variants. In the study by Boender et al. [5], it was hypothesized that this marked difference is due to a bias caused by analysing the pattern for the Netherlands only, whilst it is in fact a part of a larger pattern that extends into Belgium and Germany. As the 2023 epidemic pattern analysed here did not (yet) extend beyond the Dutch borders, we do not expect such a bias to occur in the present analysis.

Also noted before was the different shape of the kernel found for Belgium-2006 after 24 August 2006, when transport zoning within Belgium was terminated, i.e., when transports within Belgium were not subject to restrictions anymore. At present it remains an unanswered question why the lack of transport zoning restrictions in 2023 in the Netherlands did not lead in our current analysis to a kernel shape departing noticeably from those of the Germany-2006 and EU-2007 areas and periods in which a 20 km transport zoning was (mostly) in place. To further study the potential effect of differences in zoning, as suggested by a recent analysis [23], a more detailed parameterization of the transmission kernel can be used. This Levy-flight parameterization includes a parameter that can be mechanistically interpreted as a range of movement restriction. For the BTV epidemics in 2006 this parameter took values of around 25 km and 173 km in Germany and Belgium, respectively. This was in good correspondence with movement restriction zones of 20 km that were imposed in Germany and the fact that in Belgium the total country was declared one single transport zone as from 24 August. However, applying the Levy-flight parametrization to our current Netherlands-2023 dataset produced a worse fit (significantly higher AIC value) than for the parameterization of Equation (1). This may be explained by the fact that the main pattern of spread until mid-October did not yet extend beyond the Dutch borders; therefore, information is lacking on the effect of these borders as transport zone limits on the distance dependence of the transmission probabilities. In the context of BTV-8 spread in France in 2007, Courtejoie et al. [24] performed a modelling study on the role of animal movement and the effect of movement restrictions in mitigating the long-distance BTV spread. In their analysis, detailed information on animal movements between French cantons was used to obtain an attribution between animal and midge movements based on BTV transmission routes. Their analysis indicated that host movements between distant pastures of the same farm had a major contribution to BTV spread to disease-free areas. The roles of animal and vector movements have also been explored in the context of Eastern England by Turner et al. [25]; they concluded that animal movement restrictions are effective in reducing the outbreak size.

In conclusion, we analysed the between-holding transmission by estimating the spatial transmission kernel and by comparing it to transmission kernels estimated earlier for BTV-8 transmission in Northwestern Europe in 2006 and 2007. We found that the 2023 BTV-3 transmission kernel has a long-distance spatial range (across tens of kilometres), demonstrating that, in addition to the short-distance dispersal of infected midges, other transmission routes such as livestock transports may play an important role. The 2023 BTV-3 kernel parameters are found to be in line with those of the transmission kernel estimated previously for the between-holding spread of BTV-8 in Germany in 2006 and Europe in 2007.

## Figures and Tables

**Table 1:** Kernel parameter estimates obtained for BTV-3 transmission in the Netherlands in the period from 31 August to 17 October 2023, compared to estimates obtained previously [5,18] for BTV-8 in 2006–2007.

BTV Epidemic Dataset	*λ*_0_ (10^−6^ day^−1^)	*α*	*r*_0_ (km)	Reference
BTV-3 Netherlands-2023	22 (19, 25)	2.9 (2.7, 3.2)	26 (22, 29)	This study
BTV-8 Germany-2006	9.2 (6.6, 13.4)	3.2 (2.9, 3.7)	18.0 (13.5, 23.0)	[18]
BTV-8 Europe-2007 without 2006 infected area	3.2 (2.9, 3.8)	2.6 (2.57, 2.62)	21.8 (19.5, 24.3)	[5]
BTV-8 Europe-2007 without 2006 infected farm areas	8.3 (7.2, 9.5)	2.9 (2.8, 3.0)	22.5 (20.2, 24.9)	[5]

## Data Availability

Data used for this study were made available from the Netherlands’ Food and Consumer Product Safety Authority and RVO. Restrictions are applicable to the availability of these data, and the data were used under license and are not publicly available because of privacy restrictions linked to individual farm locations. Data are possibly available from the authors upon reasonable request and with the permission of the Netherlands’ Food and Consumer Product Safety Authority and RVO.
